# A Sensitive and Fast microRNA Detection Platform Based on CRlSPR-Cas12a Coupled with Hybridization Chain Reaction and Photonic Crystal Microarray

**DOI:** 10.3390/bios15040233

**Published:** 2025-04-05

**Authors:** Bingjie Xue, Bokang Qiao, Lixin Jia, Jimei Chi, Meng Su, Yanlin Song, Jie Du

**Affiliations:** 1 Beijing Anzhen Hospital, Capital Medical University, Key Laboratory of Remodeling-Related Cardio-Vascular Diseases, Ministry of Education, Beijing Collaborative Innovation Centre for Cardiovascular Disorders, Capital Medical University, Beijing Institute of Heart, Lung and Blood Vessel Disease, No. 2 Anzhen Road, Chaoyang District, Beijing 100029, China; f14sh_0528@mail.ccmu.edu.cn (B.X.); bokang_qiao@ccmu.edu.cn (B.Q.); lixinjia@mail.ccmu.edu.cn (L.J.); 2 Institute for Biological Therapy, Henan Academy of Innovations in Medical Science, Zhengzhou, 451163, China; 3 Key Laboratory of Green Printing, Institute of Chemistry, Chinese Academy of Sciences (ICCAS)/Beijing Engineering Research Center of Nanomaterials for Green Printing Technology, Beijing National Laboratory for Molecular Sciences (BNLMS), Beijing 100190, China; chijimei@iccas.ac.cn (J.C.); sumeng1988@iccas.ac.cn (M.S.); 4 University of Chinese Academy of Sciences, Beijing 100049, China

**Keywords:** microRNA detection, isothermal, CRISPR/Cas12a, hybridized chain reaction, one-pot, photonic crystal

## Abstract

Changes in microRNA (miRNA) levels are closely associated with the pathological processes of many diseases. The sensitive and fast detection of miRNAs is critical for diagnosis and prognosis. Here, we report a platform employing CRISPR/Cas12a to recognize and report changes in miRNA levels while avoiding complex multi-thermal cycling procedures. A non-enzyme-dependent hybridization chain reaction (HCR) was used to convert the miRNA signal into double-stranded DNA, which contained a Cas12a activation sequence. The target sequence was amplified simply and isothermally, enabling the test to be executed at a constant temperature of 37 °C. The detection platform had the capacity to measure concentrations down to the picomolar level, and the target miRNA could be distinguished at the nanomolar level. By using photonic crystal microarrays with a stopband-matched emission spectrum of the fluorescent-quencher modified reporter, the fluorescence signal was moderately enhanced to increase the sensitivity. With this enhancement, analyzable fluorescence results were obtained in 15 min. The HCR and Cas12a cleavage processes could be conducted in a single tube by separating the two procedures into the bottom and the cap. We verified the sensitivity and specificity of this one-pot system, and both were comparable to those of the two-step method. Overall, our study produced a fast and sensitive miRNA detection platform based on a CRISPR/Cas12a system and enzyme-free HCR amplification. This platform may serve as a potential solution for miRNA detection in clinical practice.

## 1. Introduction

As small non-coding RNAs, microRNAs (miRNAs) are released extracellularly and circulate in a protected form in bodily fluids, making them potentially valuable biomarkers. Moreover, miRNAs have advantages as biomarkers for ischemic heart disease; they are detectable in the blood, have relatively high expression levels, and can be measured in blood samples using a simple and cost-effective qPCR quantification method [1]. Additional advantages of non-coding miRNAs as biomarkers include their easy detection in body fluids, cell type-specific expression patterns, and fluctuation of expression levels in response to stress or disease [2].

Small RNAs cannot be detected using conventional RT-qPCR (real-time quantitative polymerase chain reaction) methods, owing to their short length. Currently, commercial PCR kits for miRNA detection that employ bulge-loop method or poly(A) tailing method are available, but both methods still have shortcomings, including large sample requirements and relatively low specificity. Multiple miRNAs can be detected in the blood after a heart attack, but current detection techniques are time consuming and fail to meet the rapid diagnostic needs of heart attack patients [3]. The detection of circulating non-coding RNAs is not yet applied in clinical practice and is not included in guidelines, owing to a lack of standardized procedures for extracting circulating nucleic acids from samples, a limited yield, and difficulty in quantification [4]. High-performance detection of miRNAs in biological samples poses a challenge owing to their varying chain lengths, highly similar sequences within the same family, and significant differences in content, origin, and carriers among different biological samples. There is an urgent need for super-sensitive, specific, and robust bioanalytical methods and sensors to advance the development of clinically applicable miRNA biomarkers [5].

The CRISPR/Cas system with collateral activity can be utilized for sensitive and efficient nucleic acid detection and has clinical translational potential. Researchers have realized the detection of a variety of viruses [6,7,8,9,10,11], and the system is being further developed for the detection of miRNA disease markers [12,13]. The signal transformation and amplification of target miRNAs by CRISPR/Cas combined with enzyme-free isothermal hybridization chain reaction (HCR) have been reported to achieve accurate and convenient detection of miRNAs [14,15].

In 2004, Dirks and Pierce [16] first introduced the concept of HCR, in which stable DNA monomers assemble only upon exposure to a target DNA fragment, serving as a triggered amplification methodology. This process allows DNA to act as an amplifying transducer for biosensing applications. In the simplest version of this enzyme-free reaction process, two stable species of DNA hairpins coexist in solution until the introduction of initiator strands triggers a cascade of hybridization events that yields nicked double helices analogous to alternating copolymers. In the absence of the initiator strands, the hairpins do not interact, ensuring selective triggering by the initiator strands. The designed DNA hairpin pairs self-assemble into a long-nicked DNA duplex and can be employed to amplify target sequences. Hai-Yan Jia et al. reported a miRNA detection platform based on CRISPR/Cas12a combined with HCR in which the HCR circuit is used to convert miRNA signals into double-stranded DNA activators detectable by Cas12a. After activation, Cas12a continuously cuts the fluorescent reporters, producing a multiplied fluorescence signal [17].

In this study, we combined CRISPR/Cas12a with the HCR enzyme-free isothermal amplification strategy. The HCR process was initiated by the target miRNA. The double-stranded DNA activator of Cas12a was amplified as the product of HCR. As Cas12a/crRNA combined with its activator, the single-stranded DNA reporters (F-Q) modified by the fluorescent group 6-carboxyfluorescein (FAM) and the quenching group (BHQ1) on each end were unlocked by trans-cleavage to achieve fluorescence signal output. The fluorescence intensity was further enhanced by an inkjet-printed hydrophilic photonic crystal microarray (polystyrene nanospheres) on the hydrophobic polyethylene terephthalate (PET) substrate. To match the stopband of the photonic crystal, the fluorescent groups of the reporters were changed into Cyanie5 (Cy5), and a 4.9-fold enhancement was achieved due to the photonic band-gap property and the enrichment by the hydrophilic–hydrophobic microarray. Furthermore, we managed to integrate the two isothermal reactions into one tube and maintained comparable sensitivity and specificity to the original two-step process, shortening the total turnover time of the HCR-CRISPR assay and increasing its potential for translational applications.

## 2. Materials and Methods

### 2.1. Materials and Reagents

NEBuffer™ r2.1 and EnGen^®^ Lba Cas12a (Cpf1) were purchased from New England Biolab (Ipswich, MA, USA). All the nucleic acid strands, including miRNA, crRNA, hairpins, and F-Q reporters, were synthesized by TSINGKE (Beijing, China). DNA ladders and DEPC H_2_O were purchased from Sangon (Shanghai, China). ExRed was purchased from ZOMANBIO (Beijing, China). Agarose, TAE buffer, and all other chemical reagents were purchased from Solarbio^®^ Life Sciences (Beijing, China).

### 2.2. Instrumentation

Isothermal incubation and fluorescence detection were performed by BioRad CFX96 (Hercules, CA, USA). Photonic crystal microarrays were scanned by CapitalBio^®^ LuxScan 10K/A (Beijing, China). Gel electrophoresis results were visualized on a gel imaging analyzer (Beijing, China).

### 2.3. Preparation of Hairpins and HCRs

Each kind of DNA hairpin was incubated in buffer [17] containing 140 mM NaCl, 5 mM KCl, and 20 mM Tris-HCl (pH = 7.2) for annealing into a stem-loop hairpin-like secondary structure to ensure that the hairpin monomers remained in the closed stem-loop state before HCR and to avoid triggering non-specific hybridization.

### 2.4. CRISPR/Cas12a Cleavage

Using nuclease-free tubes and reagents. The crRNA was diluted with DEPC H_2_O. DEPC H_2_O, buffer r2.1 (10×), crRNA (1 μM), and Cas12a (1 μM) were pre-mixed at a ratio of 7:1:1:1 [12] and incubated at room temperature (RT; approximately 25 °C) for 10 min. Detection tests were prepared by combining every 16 μL pre-mixture with 2 μL HCR products and 2 μL F-Q reporters (10 μM), for a total volume of 20 μL. The qPCR equipment was set at 37 °C, and the samples were incubated for 30 min. Fluorescence signals were read every 30 s.

For the one-pot reactions, the 15 μL HCR portion was prepared at the bottom of the tube, while the 15 μL CRISPR portion was added to the cap of the tube. First, the entire tube was immersed in a metal bath and incubated at 37 °C for 30 min. Then, the tube was inverted and spun quickly to thoroughly mix the two portions. Finally, the 37 °C incubation process was continued, and the fluorescence signals were read every 30 s.

### 2.5. Detection on Printed Photonic Crystal Microarrays

As previously described [18], a photonic crystal microarray is prepared by inkjet printing on hydrophobic plastic substrates self-assembled from polystyrene nanospheres. The tailored microarray is fixed onto the glass slide to fit the scanner. During this detection process, the FAM F-Q reporters were replaced by Cy5 F-Q reporters, which were added onto the photonic crystal microarray after incubation at 37 °C for 15 min in the prepared CRISPR-HCR system. The detection volume of each array was 2 μL. The slide was placed on the surface of the metal bath heating table for 5–15 min of incubation and then sent to a LuxScan scanner for fluorescence image capturing. ImageProPlus was used for statistical analysis of grayscale values.

### 2.6. Gel Electrophoresis Analysis

To analyze the HCR products, 2% agarose gel electrophoresis was performed in 1× TAE buffer conducted at a constant voltage of 120 V for 30 min. The gel was pre-stained with ExRed.

## 3. Results and Discussion

### 3.1. Principles of miRNA Detection

**Figure 1:**
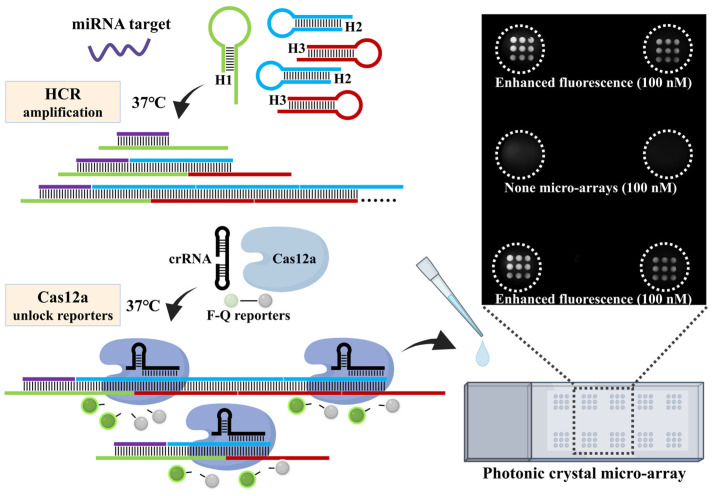
Schematic illustration of miRNA detection by CRISPR/Cas12a and enhanced fluorescence by photonic crystal microarrays.

The strategy of coupling CRISPR-Cas12a and HCR for miRNA detection is illustrated in Figure 1. This strategy employed DNA hairpin 1 (H1) containing a miRNA recognition sequence and two universal DNA hairpins, namely, DNA hairpin 2 (H2) and DNA hairpin 3 (H3). When the target miRNA is present, the stem-loop structure of H1 becomes open due to the annealing of miRNA targets and then induces the alternate hybridization of H2 and H3, finally forming a nicked DNA duplex containing several Cas12a activation sites. Thus, the trans-cleavage of CRISPR-Cas12a is activated, and the F-Q probe is cleaved indiscriminately, ultimately producing a fluorescence signal. When the target miRNA is absent, the HCR is not triggered; this prevents the trans-cleavage of Cas12a and the subsequent cleavage of the F-Q probe, and no fluorescence signal is generated. Double-stranded DNA activators generated by enzyme-free amplification are recognized and cleaved by Cas12a, reducing the risk of contaminating subsequent detections. The trans-cleavage of the fluorescence reporters by activated Cas12a/crRNA is a multi-turnover reaction, and the photonic crystal microarray enhances the fluorescence signal. As a result, the reaction time is reduced, making the detection process more rapid and convenient.

### 3.2. Feasibility of the Detection Strategy

**Figure 2:**
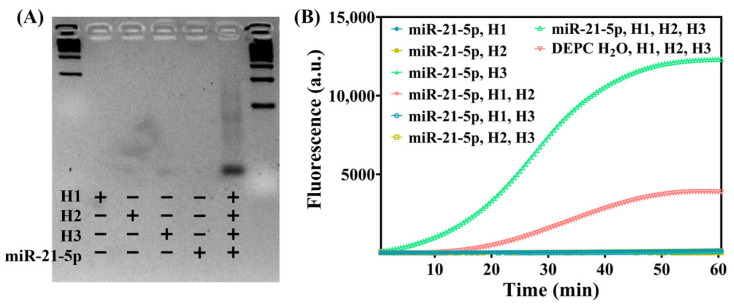
(**A**) Gel electrophoresis of HCR products. Components in each lane: 500 bp DNA ladder; H1 3 μL + DEPC H_2_O 12 μL; H2 5 μL + DEPC H_2_O 10 μL; H3 5 μL + DEPC H_2_O 10 μL; H1 3 μL + H2 5 μL + H3 5 μL + DNA mimics of miR-21-5p 2 μL; 100bp DNA ladder. (**B**) Real-time fluorescence curves of HCR-CRISPR detection of miR-21-5p.

First, 2% agarose gel electrophoresis was used to validate the effective initiation of HCR by miR-21-5p (Figure 2A). To ensure that RNA degradation during the electrophoresis process did not affect the detection results, a DNA mimic of miR-21-5p was used, in which uracil (U) in the sequence was replaced with thymine (T). The different lanes of the gel were loaded with mixtures containing different components, and the gel was incubated at 37 °C for 30 min. The concentration of both the DNA hairpins and the miR-21-5p DNA analog was 1 μM. The gel imaging results demonstrated that the HCR occurred only when both the miR-21-5p DNA analog, as the detection target and HCR initiator, and the three types of DNA hairpins were present in the system. This was indicated by distinct trailing bands in corresponding lanes, suggesting the presence of multiple DNA chains with varying lengths. These products aligned with HCR reactions in which multiple initiations may occur simultaneously but progress at different rates. In other lanes, in which the components were not present in sufficient quantities, no detectable chain extension occurred, and only free short hairpins (48 nt in length with a stem up to 18 nt) were present, without any obvious bands.

Next, the CRISPR/Cas12a system was validated. First, the HCR process was used for signal conversion and amplification to convert miRNA into double-stranded DNA, which can be recognized by Cas12a. The diluted miRNA standard samples with each annealed DNA hairpin and DEPC H_2_O were prepared as follows: 3 μL H1 + 5 μL H2 + 5 μL H3 + 2 μL miRNA sample, resulting in a total volume of 20 μL for the detection system. The mixture was incubated at 37 °C in a constant temperature metal bath for 30 min. Then, 2 μL of the completed HCR product was added to a mixture containing LbaCas12a pre-incubated at RT (approximately 25 °C) for 10 min along with its crRNA (both final concentrations were set to 1 μM) and a reporter probe (final concentration, 1 μM). For blank control samples, DEPC H_2_O was used instead of miRNA. After the system was prepared, it was placed directly into a qPCR instrument for incubation at a constant temperature of 37 °C. Real-time fluorescence was detected every 30 s for 30 min. The real-time fluorescence curve (Figure 2B) demonstrated that the fluorescence signal was amplified only when all of the components were present. Since, theoretically, each miRNA during the HCR process corresponds to the generation of several double-stranded DNA sequences that activate Cas12a collateral activity upon recognition and binding, each activated Cas12a continuously cleaves single-stranded DNA reporter probes modified with fluorescent-quencher groups (F-Q), resulting in a rapid increase in fluorescence intensity. The crRNA sequence was based on previous reports [19,20,21,22], and the overall chain length was designed to facilitate in vitro chemical synthesis.

Furthermore, miR-21-5p, miR-1, and miR-208a, which have been reported as biomarkers for cardiovascular diseases [23,24,25,26,27], were selected for CRISPR-HCR detection. The peak fluorescence intensity of each CRISPR-HCR detection process was compared and analyzed (Figure S1). The results demonstrated that modifying the corresponding sequence of hairpin H1 to complement the target sequence enables the direct application of this detection platform to other short RNAs or DNAs with a chain length of 20–22 nt. Gel electrophoresis experiments (Figure 2A) confirmed that DNA analogs could also trigger HCR. Among the H1 hairpins with different chain lengths, H1-2, the one with a moderate length, exhibited the highest signal-to-noise ratio and achieved optimal detection performance. This is speculated to be because the process of opening the stem-loop-like secondary structure formed by hairpin H1-2 with a moderate chain length exhibits superior dynamics.

### 3.3. Investigation of Sensitivity and Specificity

**Figure 3:**
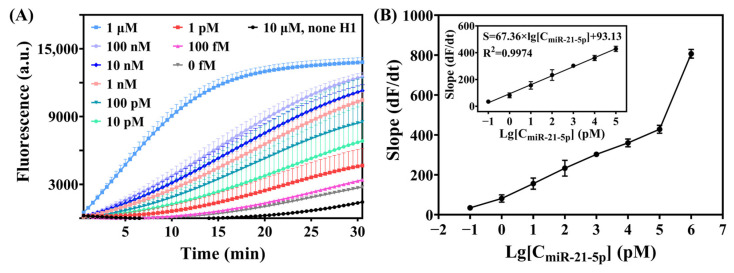
(**A**) CRISPR-HCR was used to establish the real-time fluorescence curves of miR-21-5p across a concentration gradient. After 15 min of reaction, the real-time fluorescence curve of the miRNA at 1 nm could be clearly distinguished from that of the blank control group. (**B**) Calibration curve of the logarithm of miR-21-5p concentration from 100 fM to 1 μM. The inset shows the linear relationship between fluorescence intensity and the logarithm of miR-21-5p concentration from 100 fM to 100 nM: S = 67.36 × lg[C_miR-21-5p_] + 93.13 (R^2^ = 0.9974). Each symbol and bar stand for the mean and the standard error of mean, *n* = 4.

To test the sensitivity of this detection strategy, we compared the real-time fluorescence curves of miR-21-5p at different concentrations under optimal experimental conditions. The real-time fluorescence curves (Figure 3A) showed that miR-21-5p at 1 pM could be clearly distinguished from the blank control group after 20 min of reaction. The fluorescence intensity linearly increased for 15 min. The relationship between the slope of the fluorescence intensity and the logarithm of miR-21-5p concentration (lg[C_miR-21-5p_] (pM)) was examined (Figure 3B); the slope linearly increased with the logarithm of miR-21-5p concentration over a wide range, from 1 pM to 100 nM. The linear regression equation was S = 67.36 × lg[C_miR-21-5p_] + 93.13 (R^2^ = 0.9974), with a limit of detection of 1 pM (final concentration), and the dynamic detection range of CRISPR-HCR was from 1 pM to 1 μM.

**Figure 4:**
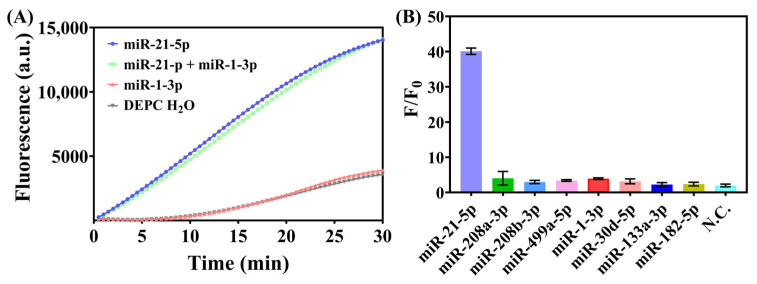
Specificity verification by testing a mixture of several miRNAs. (**A**) Real-time fluorescence curves for the mixture of target miRNA (miR-21-5p) and non-target miRNA (miR-1-3p). (**B**) Corresponding F/F0 values of miR-21-5p and other miRNAs reported to be potential biomarkers for cardiovascular diseases. The fluorescence intensity of each test was analyzed after 30 min of Cas12a cleavage. The concentrations of the miRNAs used as samples were all 1 μM.

The target miRNA and a non-target miRNA were mixed to test the specificity of the system. Our detection platform was capable of distinguishing the target miRNA with unaffected signal intensity (Figure 4A). According to the design principle, H1 is target specific and is responsible for the recognition specificity of the entire detection platform. The HCR is only initiated when a complementary miRNA that can open its stem-loop structure is present in the system. Figure 4 demonstrates that H1 for miR-21-5p selectively initiated the reaction only in samples containing miR-21-5p, and the real-time fluorescence curve for the mixed sample appears to be almost the same as the curve for the simple target miR-21-5p, indicating good specificity. When only a non-matching miRNA (miR-1-3p) was present, the detection result was similar to that for the blank control (DEPC H_2_O), suggesting that short nucleic acids not complementary to hairpin H1 do not trigger additional HCRs. We also assessed the specificity by testing different miRNAs under the same conditions, and only miR-21-5p as the target could trigger a significant fluorescence signal (Figure 4B). Further validation of detecting biospecimen would strengthen the value of the HCR-CRISPR, and we need to improve the strategy by integrating it with a suitable sample pre-processing

### 3.4. Detection on Photonic Crystal Microarrays for Enhanced Fluorescence

**Figure 5:**
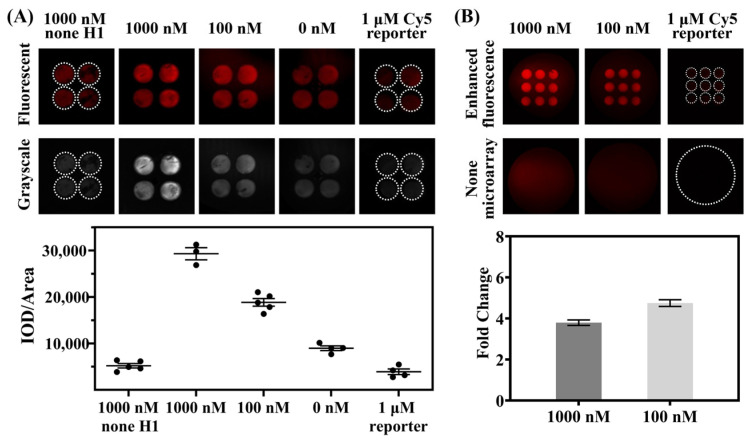
CRISPR-HCR enhanced by photonic crystal microarrays. (**A**) Representative fluorescence (red as a false color) and corresponding grayscale images acquired for the detection of miR-21-5p (top); the fluorescence intensity was analyzed from the grayscale images (bottom). (**B**) Comparison of representative fluorescence images of the detection drops with or without photonic crystal microarrays (top) and fold change in the fluorescence intensity (bottom).

By utilizing inkjet-printed photonic crystal microarrays, specific fluorescence can be selectively enhanced [18,28,29]. The prepared photonic crystal microarray for detection, as shown in Figure S2A, was matched with its stopband by replacing the previously used FAM F-Q reporter with a Cy5 F-Q reporter. After incubating the prepared CRISPR-HCR system at a constant temperature of 37 °C for 10 min, the system was dropped onto the photonic dot array and placed on a metal bath heating platform for further incubation. Once the liquid components were almost completely dry, a LuxScan scanner was used to capture fluorescence images (Figure 5). Statistical analysis of grayscale values showed that the photonic crystal microarray moderately enhanced the fluorescence intensity, which remained correlated with the target miRNA concentration (Figure 5A), and the best fold change in the fluorescent intensity was calculated to be 4.9 (when the concentration of miR-21-5p was 100 nM).

Transferring the temperature-controlled incubated droplets onto the surface of the photonic crystal microarray for continued heating allowed for continuous detection of the reaction. As water evaporates and concentrates, the system’s volume is reduced, and fluorescent groups are brought closer to the densely arranged surface of the photonic crystal array. This enrichment, together with the bandgap effect of the photonic crystal, enhanced fluorescence to 4.9 folds. As shown in Figure 5, even in the control group without the added miRNA, minimal background noise due to fluorescence enhancement by the photonic crystal was observed, indicating that the Cy5 F-Q reporter needs to be optimized. In addition, due to the viscous components of r2.1 buffer and Cas12a dilution buffer used in Cas12a pre-mixture would affect the charge characteristics of the surface of the photonic crystal and impair the band-gap effect, and given that there is research reporting that the r2.1 buffer itself can also impact the fluorescence intensity of fluorescent groups [30], the optimal fluorescence enhancement effect of the photonic crystal cannot be achieved under the current conditions for CRISPR/Cas12a. In order to fulfill the best match between the CRISPR and the photonic crystal, more design and experimentation are needed, and we will strive to improve it in future work.

### 3.5. Optimization of Experimental Operations in a Pseudo One-Pot Method

**Figure 6:**
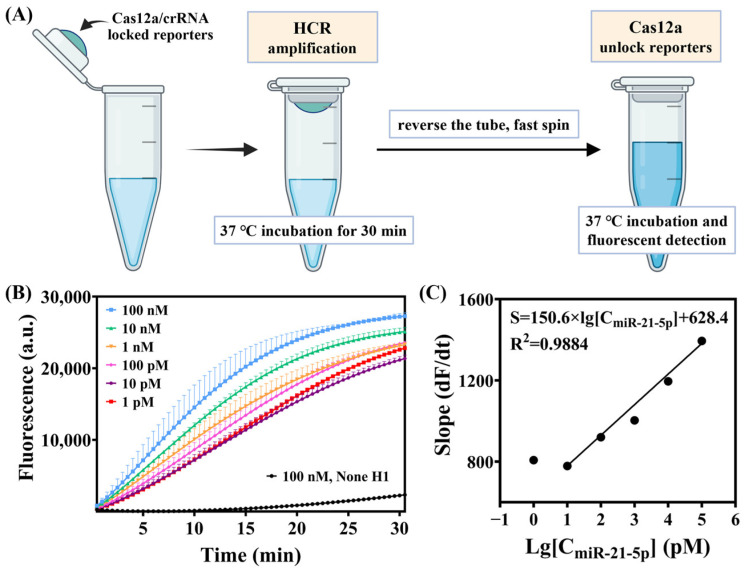
(**A**) Illustration of pseudo one-pot detection of miR-21-5p. (**B**) Real-time fluorescence curves of pseudo one-pot detection. (**C**) Calibration curve of the logarithm of miR-21-5p from 10 pM to 100 nM after incubation with Cas12a at 37 °C for 15 min.

The initial method consisted of two steps, HCR and CRISPR, both incubated at 37 °C. To simplify the overall procedure while minimizing contamination from the surrounding environment, the Cas12a/crRNA complex and FAM F-Q reporters were pre-mixed and added into the cap of the reaction tube (Figure 6). First, the HCR was incubated at 37 °C for 30 min, followed by quickly inverting and centrifuging the tube to mix the contents thoroughly. The fluorescence was then detected every 30 s for a total of 30 min while continuing incubation at 37 °C. To ensure effective cleavage of the HCR products by Cas12a in a system volume of 15 μL, we tried to gradually increase the concentration of Cas12a and crRNA in the whole system (Figure S3) and keep the concentration of Mg^2+^ at 10 mM to maintain the trans-cleavage activity [12]. The final total volume of the system was set to 30 μL according to the reference operating instructions [31].

**Figure 7:**
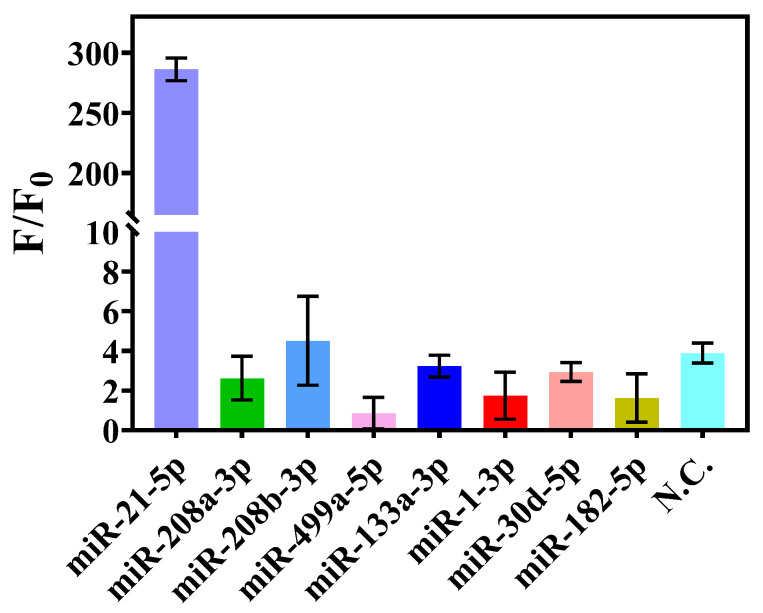
Corresponding F/F_0_ values of different miRNAs tested in the pseudo one-pot method. The fluorescence intensity of each test was analyzed after 30 min of Cas12a cleavage. The concentrations of the miRNAs used as samples were all 1 μM.

To examine the specificity of this one-pot strategy for target miRNA detection, we measured the fluorescence signal response to the target miR-21-5p and non-target RNAs (miR-208a-3p, miR-208b-3p, miR-499a-5p, miR-133-3p, miR-1-3p, miR-182-5p, miR-30d-5p, and a random sequence single strand N.C.) under the same conditions. Only when the target miR-21-5p was present did a strong fluorescence signal appear, whereas the fluorescence signals of the non-target miRNAs were very weak. The fluorescence signals of the target miRNA-21-5p and non-target RNAs differed greatly (Figure S4). Since the concentration of each component was higher than it was in the original two-step method, the F/F0 of the target increased (Figure 7).

## 4. Conclusions

In this study, we combined CRISPR-Cas12a with non–enzyme-dependent HCR amplification initiated by the target miRNA to achieve a highly sensitive and selective fluorescence signal output. The system could detect different miRNAs such as miR-21-5p, miR-208a, and miR-1 without changing the crRNA sequence under isothermal conditions. The HCR process was initiated by the target miRNA, and the double-stranded DNA activator of Cas12a was amplified as the product of the HCR. As Cas12a/crRNA combined with its activator, the single-stranded F-Q DNA reporters were unlocked by trans-cleavage to produce a fluorescence signal output. Throughout the process, cis-cleavage of Cas12a lowered the contamination risk by HCR products during subsequent detections.

The photonic crystal microarray with hydrophilic–hydrophobic micropattern is fabricated by an inkjet-printing method with simple, economical, and highly customizable operations, realizing the enrichment of the fluorescent reporter onto hydrophilic photonic crystal. The fluorescence wavelength of Cy5-BHQ2 reporters is located at the photonic crystal stopbands to augment fluorescence intensity. Combining the fluorescence enhancement and analyte enrichment, the fluorescent signal can be enhanced by the photonic crystal microarray to shorten the overall time required for the reaction to produce a detectable fluorescence signal. Analyzable fluorescence results were obtained within 15 min, demonstrating the potential of this method for clinical translational applications. When the two-step strategy adjusted into the integrated one-pot system, the overall operation process became simplified and lowered the contamination risk, while the sensitivity remained at the picomolar level, with sufficient specificity for the target miRNA.

## Data Availability

Detailed data can be obtained from the authors.

## Supplementary Materials

The following supporting information can be downloaded at: https://www.mdpi.com/article/10.3390/bios15040233/s1, Figure S1: Stem extensions of hairpin H1 from 0 to 4 nt were compared together, which provided different levels of protection to the loop from non-specific elongation; Figure S2: Characteristics of the photonic crystal microarray; Figure S3: The pseudo one-pot test of gradient concentrations of miR-21-5p in different scales of Cas12a, crRNA, buffer r2.1, and DEPC H_2_O; Figure S4: The real time fluorescent curves of miRNA-21-5p and distinct analogs; Table S1: Sequence of the oligonucleotides used in CRISPR-HCR for miRNA detection; Table S2. Sequence of the miRNA used in the specificity verification.
