# Supplementary material for: A Sensitive and Fast microRNA Detection Platform Based on CRlSPR-Cas12a Coupled with Hybridization Chain Reaction and Photonic Crystal Microarray
Source: Biosensors (Basel). 2025 Apr 5;15(4):233. doi: 10.3390/bios15040233 (PMC12024684; doi:10.3390/bios15040233)
Supplement: Supplementary file 1 [file biosensors-15-00233-s001.zip › biosensors-3462530-supplementary.pdf]

A sensitive and fast microRNA detection platform based on CRISPR-Cas12a coupled with hybridization chain reaction and photonic crystal microarray

Bingjie Xue[1], Bokang Qiao[1], Lixin Jia[1,2], Jimei Chi[3,4], Meng Su[3,4], Yanlin Song[3,4*], Jie Du[1*]

[1] Beijing Anzhen Hospital, Capital Medical University; Key Laboratory of Remodeling-Related Cardio-Vascular Diseases, Ministry of Education, Beijing Collaborative Innovation Centre for Cardiovascular Disorders, Capital Medical University, Beijing Institute of Heart, Lung and Blood Vessel Disease, No. 2 Anzhen Road, Chaoyang District, Beijing 100029, China.
[2] Institute for biological therapy, Henan Academy of Innovations in Medical Science, Zhengzhou, Henan, 451163, China.
[3]Key Laboratory of Green Printing, Institute of Chemistry, Chinese Academy of Sciences (ICCAS)/Beijing Engineering Research Center of Nanomaterials for Green Printing Technology, Beijing National Laboratory for Molecular Sciences (BNLMS), Beijing 100190, China.
[4]University of Chinese Academy of Sciences, Beijing 100049, China.

Supplementary Materials

Figures:
Figure S1: Stem extensions of hairpin H1 from 0 to 4 nt were compared together, which provide different level of protection to the loop from non-specific elongation.
Figure S2: Characteristics of the photonic crystal microarray.
Figure S3: The pseudo-one pot test of gradient concentrations of miR-21-5p in different scales of Cas12a, crRNA, buffer r2.1 and DEPC H_2O.
Figure S4: The real time fluorescent curves of miRNA-21-5p and distinct analogues; Table S1: Sequence of the oligonucleotides used in CRISPR-HCR for miRNA detection.
Tables:
Table S1. Sequence of the oligonucleotides used in CRISPR-HCR for miRNA detection
Table S2. Sequence of the miRNA used in the specificity verification

Figures

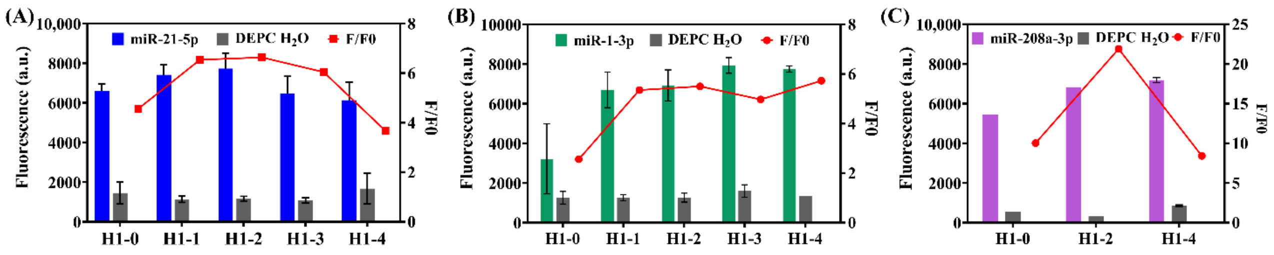

Figure S1. Stem extensions of hairpin H1 from 0 to 4 nt were compared together, which provide different level of protection to the loop from non-specific elongation. H1 with a stem extension of 2 nt (H1-2) exhibited the highest fluorescence signal response (F/F0, F and F0 are the fluorescence intensity in the present and absent of miRNA target respectively). These results indicated that stem extension of 2 nt can effectively protect the loop and be triggered to open properly by miRNA targets.

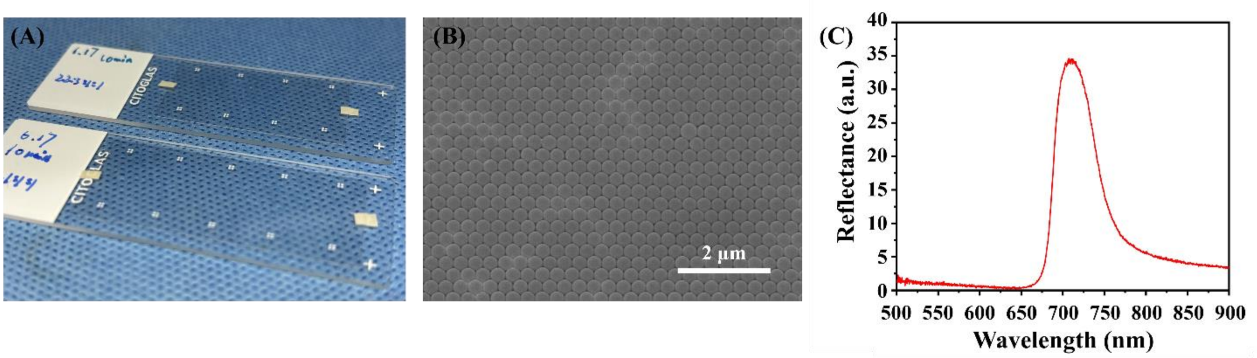

Figure S2. Characteristics of the photonic crystal microarray. (A) Macrostructure of the photonic crystal micro-arrays. (B) Microstructure characterization of the printed photonic crystal by scanning electron microscope (SEM). (C) Fluorescence enhancement of Cy5 by the photonic crystal micro-arrays.

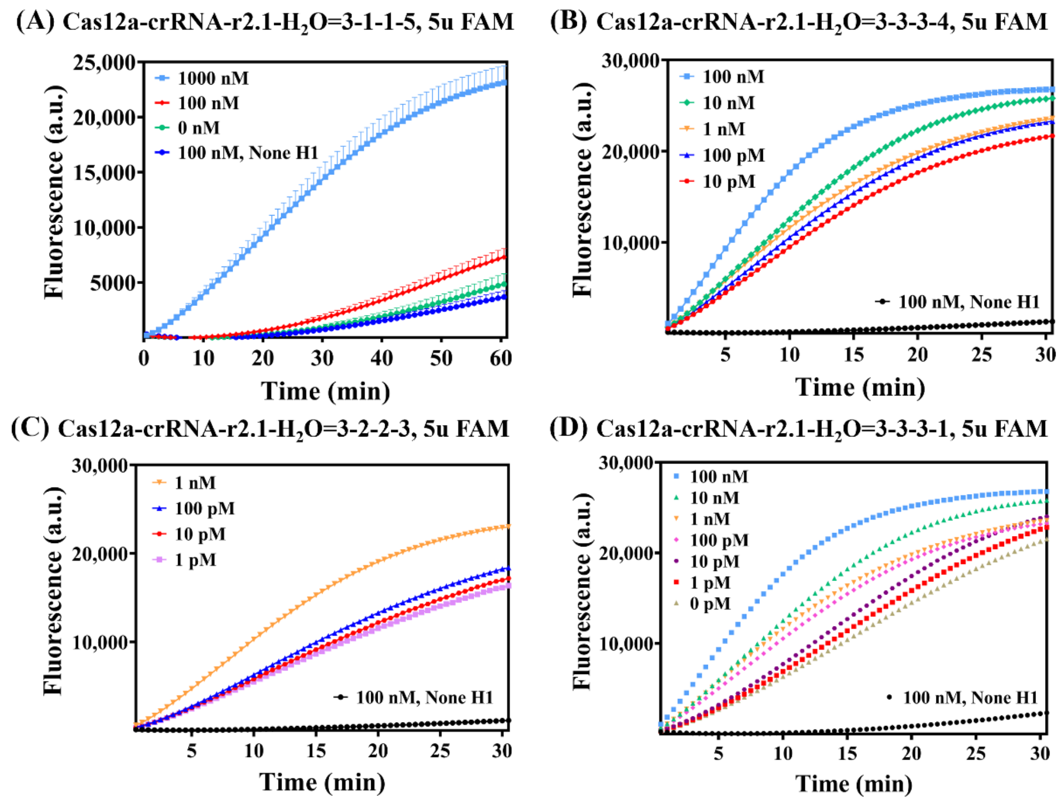

Figure S3. The pseudo-one pot test of gradient concentrations of miR-21-5p in different scales of Cas12a (1 µM), crRNA (1µM), buffer r2.1 (10×) and DEPC H_2O.

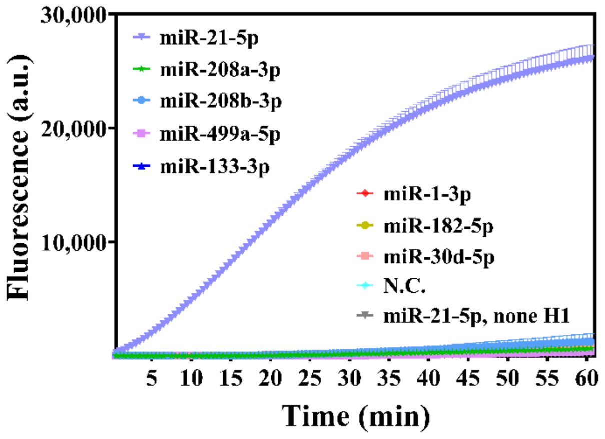

Figure S4. The real time fluorescent curves of miRNA-21-5p and distinct analogues. F/F_0 of which showed in Figure 6. The concentrations of miRNA-21-5p and miRNA analogs were all 1μM.

Table

Table S1. Sequence of the oligonucleotides used in CRISPR-HCR for miRNA detection

Name	Sequences (5'to3')
crRNA	UAAUUUCUACUAAGUGUAGAU CUAGACUACACUGAGUCUAG
H2	TTAACCAACGCAGTTTCCTAGACTCAGTGTAGTCTAGGAAACTGCGTG
H3	AGTCTAGGAAACTGCGTTGGTTAACACGCAGTTTCCTAGACTACACTG
H1-0$_{\text{miR-21-5p}}$	AGTCTAGGAAACTGCGTGGGTTAATCAACATCAGTCTGATAAGCTATTAACC
H1-1$_{\text{miR-21-5p}}$	AGTCTAGGAAACTGCGTGGGTTAATCAACATCAGTCTGATAAGCTATTAACCC
H1-2$_{\text{miR-21-5p}}$	AGTCTAGGAAACTGCGTGGGTTAATCAACATCAGTCTGATAAGCTATTAACCCA
H1-3$_{\text{miR-21-5p}}$	AGTCTAGGAAACTGCGTGGGTTAATCAACATCAGTCTGATAAGCTATTAACCCAC
H1-4$_{\text{miR-21-5p}}$	AGTCTAGGAAACTGCGTGGGTTAATCAACATCAGTCTGATAAGCTATTAACCCACG
hsa-miR-21-5p	UAGCUUAUCAGACUGAUGUUGA
FAM F-Q reporter	FAM-TTATT-BHQ
CY5 F-Q reporter	Cy5-TTATT-BHQ2

Table S2. Sequence of the miRNA used in the specificity verification

Name	Sequences (5'to3')
hsa-miR-208a-3p	AUAAGACGAGCAAAAAGCUUGU
hsa-miR-182-5p	UUUGGCAAUGGUAGAACUCACACU
hsa-miR-208a-5p	GAGCUUUUGGCCCGGGUUAUAC
hsa-miR-182-3p	UGGUUCUAGACUUGCCAACUA
hsa-miR-208b-3p	AUAAGACGAACAAAAGGUUUGU
hsa-miR-208b-5p	AAGCUUUUUGCUCGAAUUAUGU
hsa-miR-1-3p	UGGAAUGUAAAGAAGUAUGUAU
hsa-miR-499a-5p	UUAAGACUUGCAGUGAUGUUU
hsa-miR-133a-3p	UUUGGUCCCCUUCAACCAGCUG
hsa-miR-30d-5p	UGUAAACAUCCCCGACUGGAAG
